# The PhyB‐PIF4 module directly photoregulates *BR‐ENHANCED EXPRESSION* genes to enhance stem fiber cell elongation in Arabidopsis

**DOI:** 10.1111/tpj.71110

**Published:** 2026-09-14

**Authors:** Hu Xin, Fang Luo, Dan Zong, Xulei Guo, Richard D. Vierstra, Laigeng Li

**Affiliations:** ^1^ Key Laboratory for Forest Genetic & Tree Improvement and Propagation in Universities of Yunnan Province Southwest Forestry University Kunming 650224 China; ^2^ Key Laboratory of Plant Carbon Capture, CAS Center for Excellence in Molecular Plant Sciences Chinese Academy of Sciences Shanghai 200032 China; ^3^ Department of Biology Washington University in St. Louis St. Louis Missouri 63130 USA

**Keywords:** Phytochrome B, Phy‐Interacting Factors, *BR‐ENHANCED EXPRESSION* genes, photoregulation, fiber cell elongation, shade avoidance, stem growth

## Abstract

Fiber cells are a primary component of vascular tissue in angiosperms, offering mechanical support for most aerial structures, reinforcing vascular transport functions, and enabling vertical growth. Our previous research discovered light as a vital signal in modulating fiber cell‐wall thickening and thus length and strength during maturation. Here, we reveal that the red light‐sensing Phytochrome B (PhyB)/Phy‐Interacting Factor‐4 (PIF4) module plays a major photoregulatory role in Arabidopsis stem fiber growth upon activation through its negative influence on the downstream transcription factors encoded by the *BR‐ENHANCED EXPRESSION* (*BEE*) gene family. Whereas stem fiber cells in the *phyB* mutant hyperelongate, those in the *pif quadruple* mutant, which blocks the expression of *PIF1*‐*4*, are shorter. DNA binding studies showed that this PhyB/PIF module photoregulates *BEE2* expression by directly interacting with G‐box‐like elements within its promoter. Accordingly, fibers in the triple *bee123* mutant are shorter, whereas stems overexpressing *BEE2* develop longer fibers. *BEE2* in particular then encourages fiber cell elongation by directly enhancing expression of genes associated with cell‐wall loosening, two being the xyloglucan endotransglycosylases/glucanases XTH8 and XTH33. Our studies identify a key multicomponent photoregulatory cascade in fiber cell elongation, thus offering possible avenues for engineering stem properties by modulating light signals.

## Significance Statement

Accelerated stem elongation is a hallmark of the phyB/PIF‐mediated shade‐avoidance syndrome. This study reveals an R‐PhyB‐PIF4‐BEEs‐XTHs‐cell wall‐loosening circuit in stem fiber elongation, which represents the understanding of the Phy signaling cascade in controlling the shade‐avoidance syndrome in stems, offering an avenue for engineering stem growth by modulating light signals.

## INTRODUCTION

Plants stem growth begins with shoot apical meristem cells that divide, differentiate, and extensively elongate. In angiosperms, specific cell types dramatically extend and develop into fiber cells, which then become key components of the angiosperm vascular system, characterized morphologically by their elongated shape and thickened cell walls (Mauseth, 2014). They provide mechanical support for most aerial tissues, determine the shape of specific organs, enable phloem and xylem functions, and support the vertical growth of stems. As such, they represent a major renewable resource for biomass, fibers, paper, and wood (Gorshkova et al., 2012; Mikshina et al., 2013). During stem growth, xylem fiber cells in particular undergo remarkable elongation, followed by secondary cell‐wall thickening and eventual cell death (Zhu & Li, 2021), which are coordinated by various internal and external signals to ultimately define stem length (Huang et al., 2018; Zhang et al., 2024).

Fiber cell elongation is largely mediated by a host of cell wall‐loosening factors, including expansins, xyloglucan endotransglycosylases/hydrolases (XTHs), and glucanases (Cosgrove, 2005, 2024), that allow turgor pressure‐driven cell expansion/elongation. As examples, expansins such as *EXPA1*, *EXPA5*, and *EXPA7* are highly expressed in the radial expansion zones of *Populus tricocarpa*, and transgenic overexpression of *EXPA1* increases tree height and fiber cell diameter (Gray‐Mitsumune et al., 2004, 2008). Similarly, fiber‐specific upregulation of the cellulase Cel9A6 in aspen, which encodes an endo‐1,4‐β‐glucanase, increases fiber cell length in wood (Li et al., 2025; Yu et al., 2013).

Cell‐wall loosening is orchestrated in part by a complex network of internal signals working though the brassinolide (BR), gibberellic acid (GA), and auxin families of hormones (Cowling & Harberd, 1999; Evans & Cleland, 1985; Tian et al., 2022; Xiao et al., 2019). As examples, active GAs have been documented to accumulate in expanding *Populus* xylem during fiber cell elongation when the GA biosynthetic enzyme GA20ox1 is overexpressed, which in turn increases stem height and fiber length (Eriksson et al., 2000; Israelsson et al., 2005; Jeon et al., 2016). Similarly, auxin levels modulated by the KNOX family of *Populus* transcription factors, such as the Knotted‐Like Homeobox Transcription Factor (KNAT)‐5a, were found to promote xylem fiber elongation (Huang et al., 2025). As stem elongation involves the coordinated expansion of multiple tissue types (e.g., epidermis, cortex, pith, and vascular tissue), some of this regulation might involve cell‐to‐cell communications, especially between the epidermis/cortex and the interior vascular bundles (Savaldi‐Goldstein & Chory, 2008).

In Arabidopsis, BR is a crucial regulator of cell elongation, photomorphogenesis, and flowering (Bai et al., 2012; Yang et al., 2005; Zhang, Li, et al., 2013). Its signaling pathways encompass a network of transcription factors that include those encoded by the *BR‐ENHANCED EXPRESSION* (*BEE*) genes. BEE1‐3 in particular positively regulate BR signaling and promote hypocotyl elongation (Friedrichsen et al., 2002), with the BEEs family also regulating photoperiodic flowering and shade avoidance via BR–light signal coordination (Cao et al., 2022; Cifuentes‐Esquivel et al., 2013; Wang et al., 2019). Notably, constitutive overexpression of the *BEE3L* isoform driven by the CaMV 35S promoter was found to enhance stem elongation and thickening in a *Populus* hybrid, which then increased tree stem biomass (Noh et al., 2015).

Environmental cues, such as light, are also crucial to fiber cell development. Blue light, working through the CRYPTOCHROME (CRY)‐1 photoreceptor, influences stem growth, fiber cell elongation, and secondary wall thickening; accordingly, *cry1* mutants develop longer stems and fibers, while *CRY1* overexpression suppresses stem elongation (Zhang et al., 2018). Arguably, the most influential is the phytochrome (Phy) photoreceptor family working via negative control of the Phytochrome‐Interacting Factor (PIF) basic–helix–loop–helix (bHLH) family of transcriptional activators/repressors (Al‐Sady et al., 2006; Bauer et al., 2004; Lorrain et al., 2007; Quail, 2002). Phys detect red (R) and far‐red (FR) light through photointerconversion between a R‐absorbing, inactive Pr state and a FR‐absorbing biologically active Pfr state (Franklin & Quail, 2010; Quail, 2002). While Phys are inactive in darkness or under low R:FR ratios when Pfr levels are minimal, exposure to white light (WL), R, or high R:FR ratios generates high levels of active Pfr, thus enabling binding of Phys to PIFs. Formation of the PhyB‐Pfr/PIF complex then triggers degradation of both, thus depressing PIF action which in turn activates/represses downstream effectors. For fiber elongation in particular, the PhyB isoform is the dominant regulator which, upon photoactivation, leads to binding to the PIF4 isoform (Luo et al., 2022). The resulting PhyB/PIF4 complex is then degraded rapidly (Paik et al., 2019), thus activating/depressing the expression of regulators connected to cell‐wall loosening. Through this photoswitch commonly associated with the shade‐avoidance syndrome (Casal, 2012), low R:FR ratios promote fiber cell‐wall loosening, leading to elongated stems (Luo et al., 2022), but the exact molecular mechanism(s) remain unclear.

This study investigated the photoregulation of stem fiber cell elongation in Arabidopsis through PhyB and PIFs as an easily quantifiable model for light‐coordinated stem growth. Our analyses of mutant and overexpression transgenic lines, transcriptomics, and promoter studies revealed a photoregulatory circuit from the PhyB/PIF4 module to BEE2. This loop then directly activates expression of cell wall‐loosening genes, such as *XTH8* and *XTH33*, thus encouraging stem fiber elongation connected to downstream skotomorphogenic and shape avoidance responses under darkness or low Pfr levels.

## RESULTS

### Red light promotes stem elongation in Arabidopsis through PhyB and PIFs

**Figure 1:**
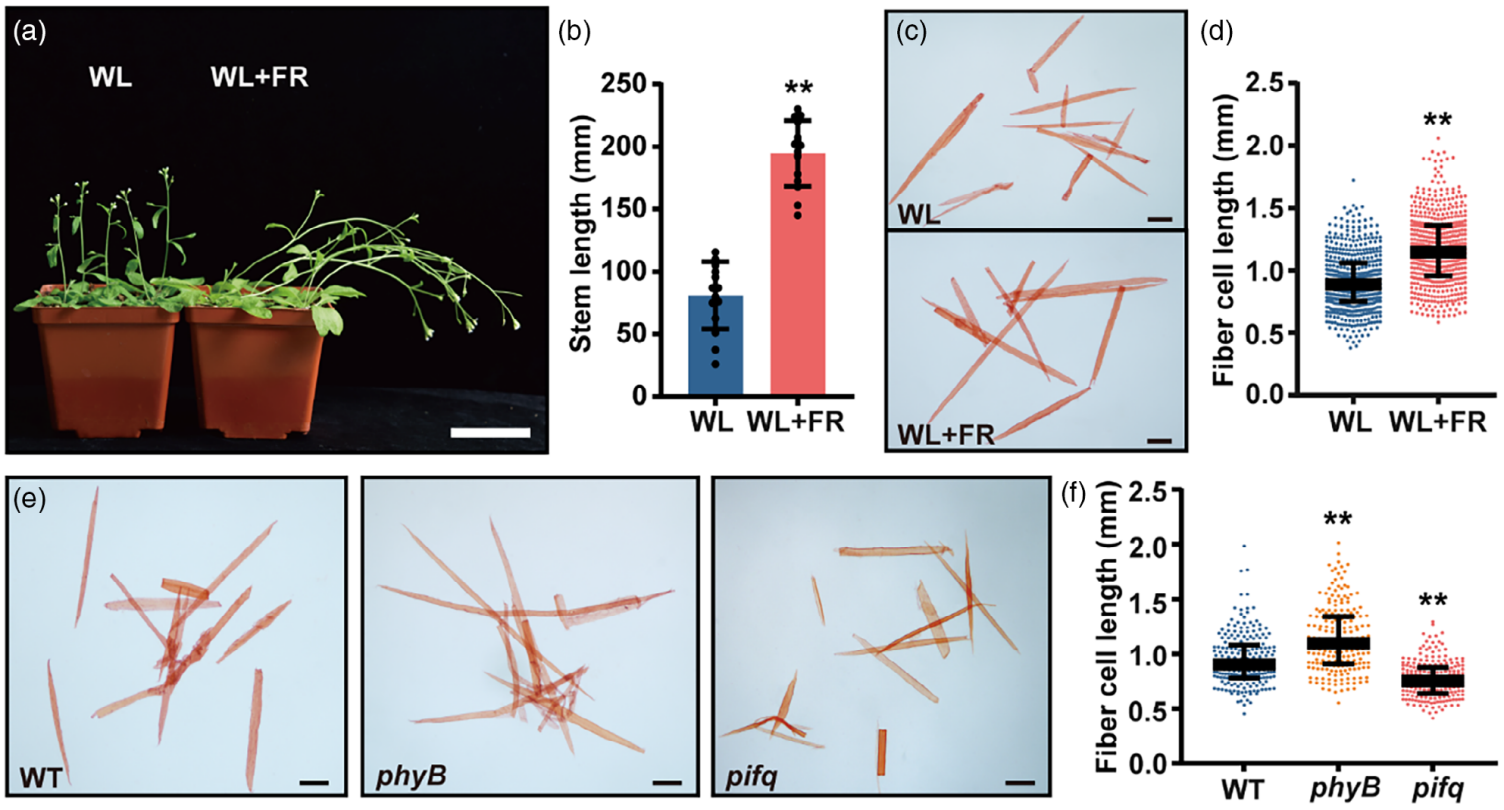
Low R:FR light environments accelerate Arabidopsis inflorescence growth and increase stem fiber cell elongation. (a) Photographs of 4‐week‐old wild‐type (WT) Arabidopsis inflorescence stems grown under white light (WL) or a low R:FR ratio. Scale bar = 5 cm. (b) Bar graphs showing the mean length of Arabidopsis inflorescence stems grown as in panel A. Data represent the mean (±SD) of 15 seedlings. (c) Photographs of representative fiber cells extracted from fully mature inflorescence stems grown as in panel (a). Cells were stained with Safranin. Scale bars = 0.2 mm. (d) Scatter plots showing the distribution of fiber cell lengths extracted from inflorescence stems grown as in panel (a). Data represent the mean (±SD) of three biological replicates each containing >100 cells. (e) Photographs of representative fiber cells extracted from fully mature inflorescence stems of the *phyB* and *pifq* mutants grown under WL, followed by staining with Safranin. Scale bars = 0.2 mm. (f) Scatter plots showing the distribution of fiber cell lengths extracted from the inflorescence stems of the *phyB* and *pifq* mutants. Data represent the mean (±SD) of three biological replicates each containing >100 cells. **Indicates significant differences among samples as determined by the Student's *t*‐test (*P* < 0.01) for panels b, d, and f.

Low R:FR ratios and thus low Pfr levels are known for promoting stem elongation, a signature feature of the shade‐avoidance syndrome (Casal, 2012; Franklin, 2008). Nevertheless, the mechanism(s) down regulating the cellular process of stem growth in response to this light regime remain inadequately understood. Utilizing the elongating inflorescences of Arabidopsis as a model, we investigated the repressive impact of Pfr on this growth. After growing the emerging stems under WL, wild‐type (WT) plants were then exposed to WL supplemented with high fluence FR to substantially reduce the R:FR ratio, thus depleting Pfr levels. As shown in Figure 1a,b, this lowered R:FR ratio significantly accelerated stem elongation and triggered their eventual collapse (also known as lodging) as the growing stems became too tall to support the inflorescence and developing flowers. Analysis of fiber cells specifically after extraction from stems using a glacial acetic acid/hydrogen peroxide mixture followed by staining of their cell walls with Safranin (Zhang et al., 2018) showed that their fiber lengths became significantly longer with the addition of FR (Figure 1c,d), indicating that low R:FR conditions enhance stem elongation in part by increasing fiber cell length.

Our previous studies on *phyB* and *pif* mutants revealed that the R‐signaling system involving the PhyB/PIF module plays a role in regulating stem elongation (Luo et al., 2022). To connect this response in stem fiber cells, we examined fiber length in the *phyB* null mutant *phyB1‐1* and in the quadruple *pifq* mutant unable to express the PIF1‐4 members within the Arabidopsis PIF family (Zhang, Mayba, et al., 2013). As shown in Figure 1e,f, the *phyB* mutant grown under WL developed significantly longer fiber cells in the stem as compared to WT, whereas the *pifq* mutant developed significantly shorter stem fiber cells, indicating that R‐FR signaling photoregulates fiber elongation through a PhyB/PIF module.

### Low R:FR Ratios induce *BEE2* expression via the PhyB/PIFs module

**Figure 2:**
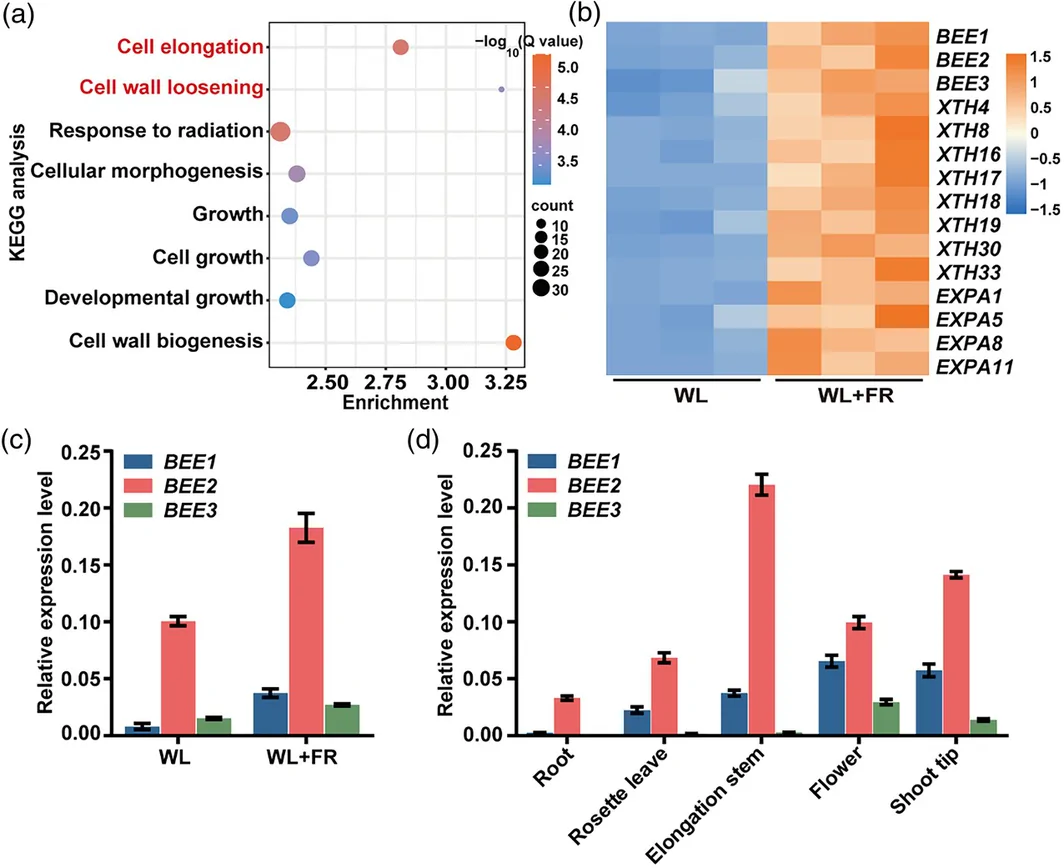
Transcriptome analysis of inflorescence stems grown under white light (WL) or a low R:FR ratio reveals photoregulated transcriptional networks. (a) Kyoto Encyclopedia of Genes and Genomes (KEGG) pathway enrichment analysis of 2541 differential expression genes (DEGs) in 4‐week‐old WT inflorescences grown in WL versus WL + FR conditions. Pathways significantly enriched based on DEGs defined by |log_2_FC| > 1 and Q‐value <0.05 are shown. Dot size indicates gene number in each category and dot color represents statistical significance (−log10[q‐value]). (b) Heatmap showing the differential expression of representative genes involved in cell elongation and cell‐wall loosening under WL versus WL + FR conditions. Shown are the data from three individual biological replicates for each light condition. Colors indicate transcript abundance normalized by Z‐score transformation across all samples for each gene. (c) Relative expression levels of *BEE1*, *BEE2*, and *BEE3* transcripts under WL and WL + FR as determined by quantitative reverse transcriptase‐polymerase chain reaction (qRT‐PCR) using total RNA isolated from WT stems. Bars represent mean (±SD) of three biological replicates normalized to that for *ACT2*. (d) Relative expression levels of *BEE1*, *BEE2*, and *BEE3* transcripts in various Arabidopsis tissues as determined by qRT‐PCR. Bars represent mean (±SD) of three biological replicates normalized to that for *ACT2*.

To elucidate how the Arabidopsis PhyB/PIF module governs inflorescence elongation, we deep sequenced the total RNA population isolated from WT stems grown either under WL or a low R:FR ratio (Figure S1A). In total, RNA sequencing (RNA‐seq) analyses of three biological replicates, processed following standard workflows, identified 20 323 transcripts commonly detected in all three replicates of stems grown under WL, and 20 106 transcripts commonly detected in those grown under WL + FR. Transcriptome comparisons identified a total of 2541 differential expression genes (DEGs) between WL‐ and WL + FR‐treated stems as defined by a Q‐value <0.05 and >2‐fold increase or >2‐fold decrease in abundance (Figure S1A,B; Table S1). Subsequent RNA profiling using the Kyoto Encyclopedia of Genes and Genomes (KEGG) database (Ogata et al., 1999) revealed that many DEGs were strongly linked to processes associated with cell elongation, cell‐wall loosening, and cell‐wall biogenesis (adjusted *P* < 0.05； Figure 2a,b; Figure S1C), which were similar to prior Arabidopsis RNA microarray data involving whole WT and *phyB* mutant seedlings exposed to FR‐enriched light environments (Devlin et al., 2003). The collective results implied that these processes were markedly perturbed by the WL + FR condition, as expected for cellular functions needed to control stem elongation and fiber cell development.

In particular, we noticed that the mRNAs encoding members of the BEE transcription factor family previously reported to play a positive role in hypocotyl elongation (Friedrichsen et al., 2002) were significantly upregulated in stems under low R:FR ratio conditions (2.9–13.3 fold increases; Figure 2b,c), thus implicating BEEs as central regulators of stem growth and fiber elongation. The Arabidopsis genome encodes three BEE paralogs, BEE1, BEE2, and BEE3 that display similar expression patterns (Toledo‐Ortiz et al., 2003). Upon examination of the corresponding mRNAs in various Arabidopsis tissues, we found that the *BEE2* transcript accumulated to a high level in the stem and showed the strongest upregulation upon exposure to a low R:FR ratio (Figure 2c,d), suggesting a pivotal function for BEE2 in stem fiber cell growth. In agreement, histochemical staining of elongating stems transgenically expressing β‐glucurosidase (GUS) under the control of the *BEE2* promoter revealed that the *BEE2* gene is expressed in interfascicular and xylem fiber cells (Figure S2).

### BEE2 promotes stem fiber cell elongation modulated by R:FR light signals

**Figure 3:**
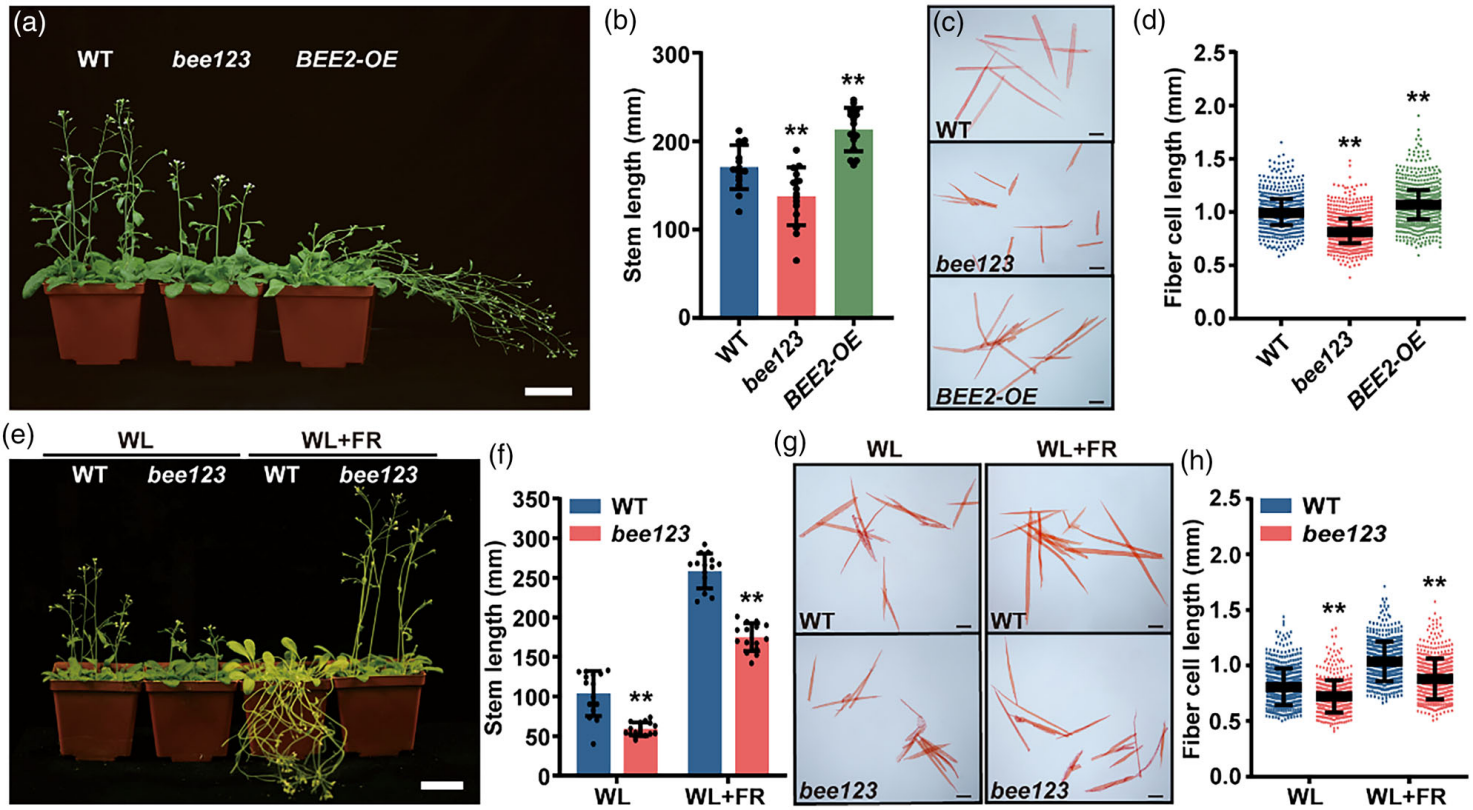
Roles of *BEEs* in stem elongation is modulated by the R:FR ratio. (a) Photographs wild‐type (WT) Arabidopsis and the *bee123*, and *BEE2‐OE* variants grown under white light (WL). Levels of BEE2‐OE protein can be found in Figure S3. Scale bar = 5 cm. (b) Bar graphs showing the mean inflorescence stem length of WT, *bee123*, and *BEE2‐OE* plants grown under the conditions shown in panel (a). Data represent the mean (±SD) of 15 seedlings. (c) Photographs of representative fiber cells extracted from inflorescence stems grown as in panel A. Cells were stained with Safranin. Scale bars = 0.2 mm. (d) Scatter plots showing the distribution of fiber cell lengths from the inflorescence stems of WT, *bee123*, and *BEE2‐OE* plants grown as in panel (a). Data represent mean ± SD of three biological replicates, each containing >100 cells. (e) Photographs of WT and *bee123* mutant grown under WL or WL + FR. Scale bar = 5 cm. (f) Bar graphs showing the mean inflorescence stem length of WT and *bee123* mutant plants grown under the conditions shown in panel (e). Data represent mean (±SD) of 15 seedlings. (g) Photographs of representative fiber cells extracted from the inflorescence stems of WT and *bee123* mutant plants grown under WL or WL + FR. Cells were stained with safranin. Scale bars = 0.2 mm. (h) Scatter plots showing the distribution of fiber cell lengths from WT and *bee123* inflorescence stems grown under WL or WL + FR. Data represent mean (±SD) of three biological replicates, each containing >100 cells. ** Indicates significant differences as determined by the Student's *t*‐test (*P* < 0.01) for the comparisons shown in panels b, d, f and h.

To help define *BEE2* function(s) in stem growth, we analyzed a *bee123* (*bee1 bee2 bee3*) triple mutant and transgenic plants overexpressing *BEE2* specifically (*BEE2‐OE*) (Figure 3a; Figure S3). The triple mutant was generated by crossing the individual homozygous *bee1‐1*, *bee2‐1*, and *bee3‐1* T‐DNA insertion mutants, which were previously described as null by Friedrichsen *et al*. (2002). The *BEE2‐OE* line was generated by transforming WT seedlings with a full‐length *BEE2* cDNA fused in‐frame to a C‐terminal hemagglutinin (HA) epitope tag (YPYDVPDYA) and constitutively expressed under the control of the CaMV *35S* promoter (Figure S3). As judged by quantitative reverse transcriptase‐polymerase chain reaction (qRT‐PCR) with *BEE2*‐specific oligonucleotides, the *BEE2‐OE* plants had approximately 10‐fold more *BEE2* mRNA than WT (Figure S3A) and showed obvious expression of the BEE2‐HA protein as determined by immunoblot analysis with anti‐HA antibodies (Figure S3B). Under WL, the *bee123* triple mutant grew significantly shorter stems as compared to the WT, whereas the *BEE2‐OE* line grew significantly longer stems with an increased frequency of lodging (Figure 3a,b). Similarly, fiber cells extracted from stems were significantly shorter in the *bee123* mutant and significantly longer in those from the *BEE2‐OE* line versus WT as judged by a Student's *t*‐test of more than 100 fiber cells each (Figure 3c,d). These results demonstrated that BEE2, and possibly other members of the Arabidopsis BEE family, promote fiber cell elongation, possibly by regulating the expression of cell wall‐loosening genes.

To determine how BEEs‐mediated regulation of stem elongation is affected by light, we grew the *bee123* triple mutant under the low R:FR ratio. The mutant plants developed shorter inflorescences than WT with a lessened frequency of lodging (Figure 3e,f). The elongation of stem fiber cells in the *bee123* mutant was also markedly reduced by the low R:FR ratio, with the fiber cells remaining significantly shorter than those of the WT based on measurements of more than 100 fiber cells (Figure 3g,h). These results indicated that the BEEs promote stem and fiber cell elongation under low R:FR ratios or possibly even darkness, which in turn lowers Pfr levels.

### BEE2 acts downstream of PIFs to promote stem fiber cell elongation

**Figure 4:**
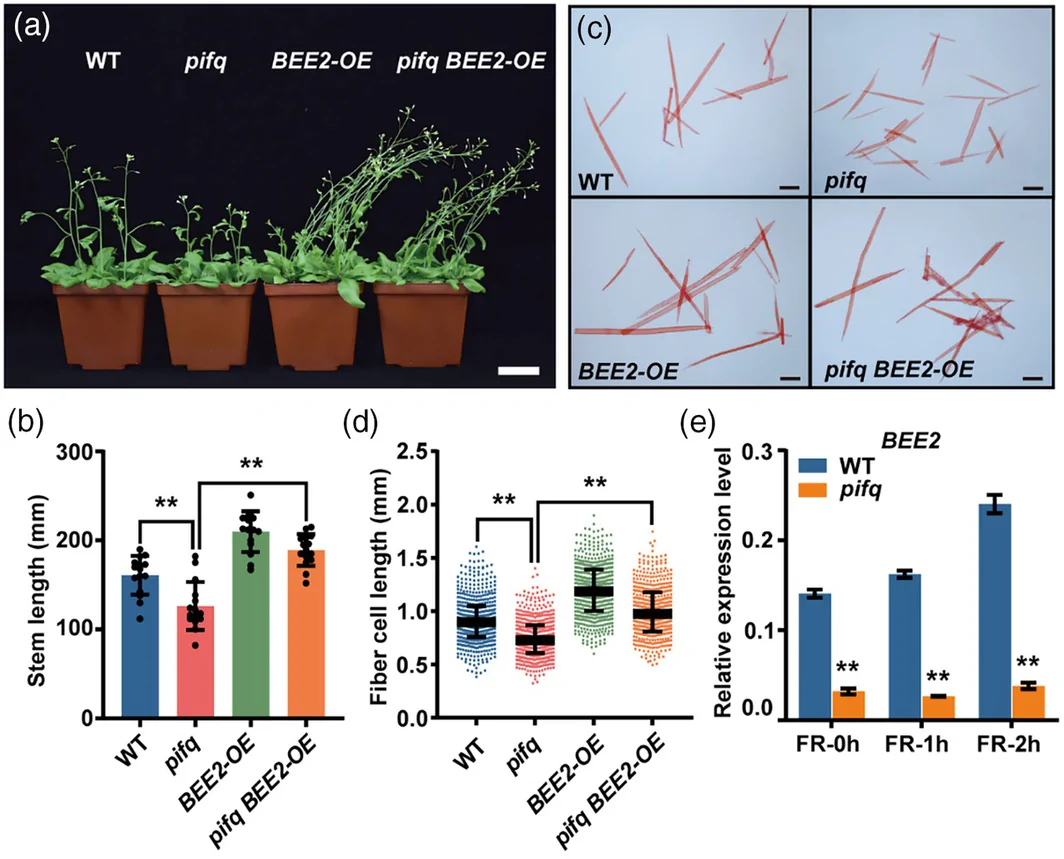
*BEE2* genetically interacts with *PIF4* in stem elongation. (a) Photographs of wild‐type (WT) Arabidopsis, the *pifq*, *BEE2‐OE*, and *pifq BEE2‐OE* variants grown under white light (WL) for 4 weeks. Scale bar = 5 cm. (b) Bar graphs showing the mean height of WT, *pifq*, *BEE2‐OE*, and *pifq BEE2‐OE* plants grown as shown in panel (a). Data represent the mean (±SD) of 15 seedlings. ** Indicates significant differences as determined by the Tukey's HSD test (*P* < 0.01). (c) Photographs of representative fiber cells extracted from the inflorescence stems of WT, *pifq*, *BEE2‐OE*, and *pifq BEE2‐OE* plants, stained with safranin. Scale bars = 0.2 mm. (d) Scatter plots showing the distribution of fiber cell lengths in WT, *pifq*, *BEE2‐OE*, and *pifq BEE2‐OE* inflorescence stems. Data represent mean (±SD) of three biological replicates, each containing >100 cells. ** Indicates significant differences as determined by the Tukey's HSD test (*P* < 0.01). (e) Relative expression levels of *BEE2* in WT and the *pifq* mutant as determined by quantitative reverse transcriptase‐polymerase chain reaction (qRT‐PCR) using total RNA isolated from 4‐week‐old stems before and after the FR light treatment. Bars are the mean (±SD) of three biological replicates normalized to that for *ACT2*. ** Indicates significant differences as determined by the Student's *t*‐test (*P* < 0.01).

Given that expression of the *BEE* genes during fiber cell elongation is responsive to R:FR cues, we studied this action genetically to understand whether these transcription factors work downstream of the PHYB/PIF signaling pathway. Here, we generated a *pifq BEE2‐OE* line by introducing the *BEE2‐HA* transgene into the *pifq* background and then tested whether *BEE2* overexpression in the *pifq* mutant could override the absence of PIF1‐4. Immunoblot analysis with anti‐HA antibodies confirmed that the BEE2‐HA fusion protein retained expression in the *pifq* background (Figure S4B). As shown in Figure 4a,b, the short‐stem phenotype of *pifq* mutants was substantially rescued by increased expression of *BEE*2. The reduced length of fiber cells in the *pifq* mutant was also significantly restored by *BEE2* overexpression, showing an approximately 1.3‐fold increase in fiber cell length under WL as compared to the response of *pifq* fibers (Figure 4c,d).

We next examined how BEE expression might be specifically connected to the PhyB/PIFs module. In WT Arabidopsis, a low R:FR ratio markedly elevated *BEE2* transcript level in stems; when the plants were transferred from WL to WL + FR, a 1.7‐fold increase in *BEE2* mRNA levels was seen after 2 h as compared with WL alone (Figure 4e). This induction was not seen in the *pifq* mutant (Figure 4e), implying that the upregulated expression of *BEE2* depends, at least in part, on PIF activity. Consequently, we concluded that the repressive PhyB/PIF4 module acts upstream of *BEE* gene expression, whose lack of turnover under low R:FR promotes *BEEs* expression, which then enables stem and fiber cell elongation.

### PIF4 directly binds to the *BEE2* promoter to activate its expression

**Figure 5:**
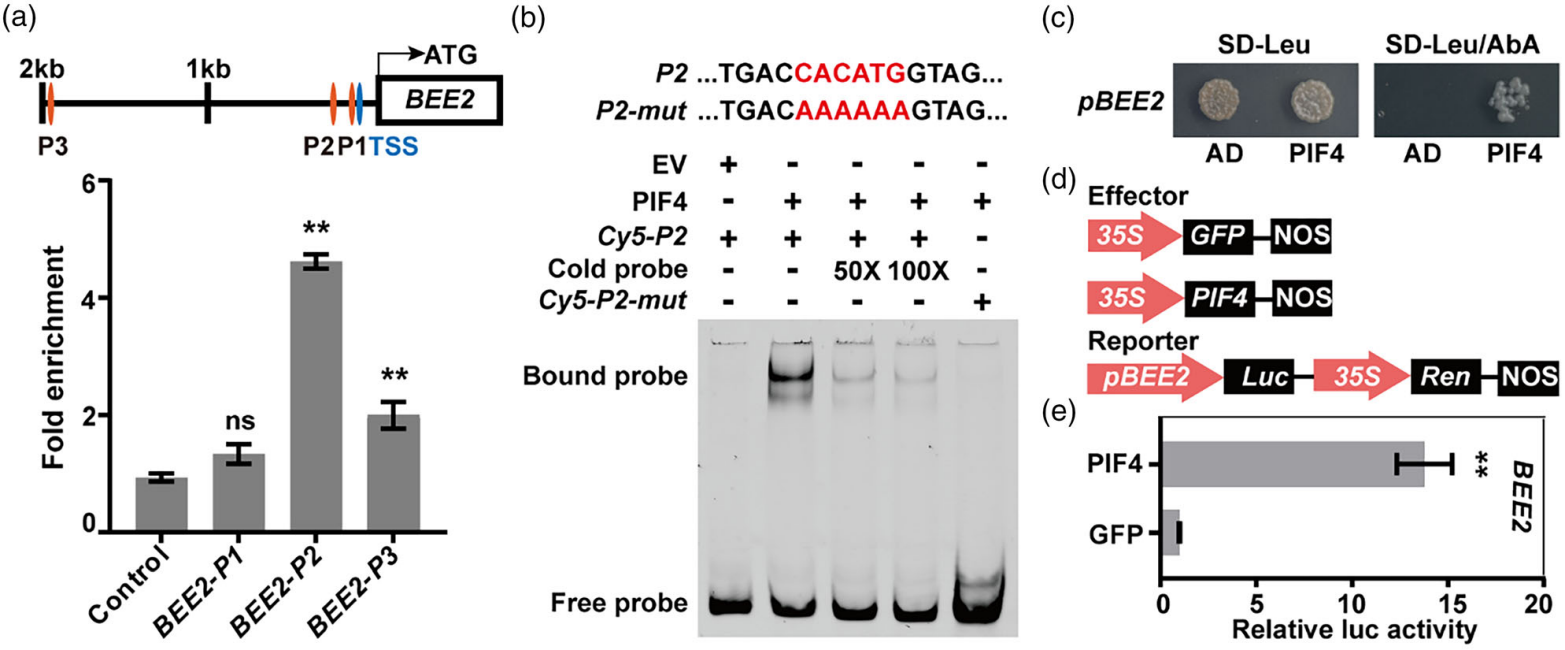
PIF4 directly binds to the *BEE2* promoter to activate its expression. (a) Upper panel: schematic diagram of the *BEE2* promoter showing the locations of the P1, P2, and P3 G‐box‐like sequences used for ChIP‐qPCR analysis. Lower panel: ChIP‐qPCR results showing the enrichment of PIF4 by each promoter fragment. Data represent the mean (±SD) of technical replicates from a representative experiment. Similar results were obtained in three independent experiments. The orange ovals in the gene diagram identify the positions of the predicted G‐box‐like elements. The predicted transcription start sites (TSS) and translation start code (ATG) are indicated. (b) Electrophoretic mobility shift assay (EMSA) showing the specific binding of the PIF4 protein to the P2 promoter fragment of *BEE2* labeled with Cy5. ‘−’ and ‘+’ indicate the absence or presence of 6xHis‐PIF4 protein, labeled probes, mutated probes, or cold competitors in the EMSA assays. Top sequences show the WT P2 and the P2‐mut DNA sequences. (c) Yeast one‐hybrid (Y1H) assays validating the interaction between PIF4 and the *BEE2* promoter. Yeast strains carrying *pBEE2‐AbAi* were transformed with the AD‐PIF4 or empty vector (AD) and grown on SD‐Leu medium or SD‐Leu/AbA selective medium. (d) Schematic diagram of the dual‐luciferase reporter (*pBEE2::LUC/REN*) and effector constructions (*35S::PIF4*, *35S::GFP*) used for transient expression assays in Arabidopsis protoplasts. (e) PIF4 activates the *BEE2* promoter in Arabidopsis protoplasts. Protoplasts from rosette leaves were co‐transformed with the reporter constructions together with different effector constructions. After transfection, the protoplasts were kept in the dark for 16 h. Relative luciferase activity (LUC/REN) was normalized to that of protoplasts transformed with the reporter and empty effector (GFP). Bars represent mean (±SD) of three biological replicates. ** Indicates significant differences as determined by the Student's *t*‐test (*P* < 0.01) for the comparisons shown in panels a and e.

To examine how the PhyB/PIF4 module might regulate *BEE2* expression, we tested whether PIF4 binds directly with the *BEE2* promoter. PIF4 had previously been shown to bind G‐box cis‐elements (Ezer et al., 2017), which have been implicated in the transcriptional regulation of hundreds of plant genes associated with growth, temperature, light, drought, and immune signaling, and metal homeostasis through binding with basic leucine zipper (bZIP) and/or bHLH transcription factors, such as PIF4 (Jakoby et al., 2002; Leivar & Monte, 2014; Toledo‐Ortiz et al., 2003). From a sequence analysis of the *BEE2* promoter, we found three possible G‐box relatives, P1, P2, and P3 with CATGTG, CACATG, and CACATG core sequences, respectively (Figure 5a; Figure S5A), that differed slightly from the consensus motif (CACGTG). Using electrophoretic mobility shift assays (EMSA) in combination with recombinant full‐length PIF4 purified based on an N‐terminal, in‐frame 6His sequence (MHHHHHH), we found that PIF4 binds to all three P1, P2, and P3 elements, with the strongest binding seen at the P2 site (Figure S5B).

We next tested for this *BEE2* promoter binding by PIF4 *in planta*. Chromatin immunoprecipitation (ChIP) assays, using DNA from plants overexpressing PIF4 in‐frame with a C‐terminal MYC epitope (EQKLISEEDL) (Ma et al., 2015), showed that the PIF4‐MYC protein was enriched at two cis‐element sites within the *BEE2* promoter—P2 (5′‐CACATG‐3′) and P3 (5′‐CACATG‐3′)—with the strongest enrichment observed at the P2 position (Figure 5a). Since the P2 sequence showed the highest affinity for PIF4‐MYC both *in vitro* and *in viv*o, we further assessed its binding specificity by EMSA using a collection of oligonucleotides harboring WT or mutant versions of the P2 G‐box labeled with the fluorescent tag Cy5 (Steiner & Pfannschmidt, 2009). Mutation (AAAAAA) of the CACATG motif in P2 abolished PIF4 binding, and competition with excess unlabeled probe markedly reduced the binding signal, confirming the binding specificity (Figure 5b).

To further confirm the EMSA and ChIP results, we used yeast one‐hybrid (Y1H) assays to test whether PIF4 could directly bind to the *BEE2* promoter *in vivo* using approximately 575‐bp fragment containing the P2 element. This fragment was cloned into the pABAi vector as the bait, and either an empty pGADT7 vector or pGADT7 expressing full‐length PIF4 tagged in‐frame with a N‐terminal HA was used as the prey. In this case, yeast colony growth under restricted growth conditions in the presence of aureobasidin A (AbA) was seen for cells also expressing the PIF4 construction but not in those transformed with the empty vector (Figure 5c).

We further tested the *PIF4:BEE2*‐promoter interaction using dual‐luciferase reporter (dual‐LUC) assays with Arabidopsis protoplasts designed to assay spectrophotometrically for *BEE2* promoter‐driven expression, using light emission from firefly luciferase (LUC) normalized to a Renilla luciferase (REN) control (Yoo et al., 2007). These Dual‐LUC assays using PIF4 constitutively expressed by the CaMV *35S* promoter showed that the PIF4 protein can significantly activate *BEE2* promoter activity. As compared to a green fluorescent protein (GFP)‐vector control (*35S::GFP*), co‐expression of *35S::PIF4* with the *proBEE2::LUC* reporter resulted in approximately 14‐fold increase in LUC activity measured by normalized luminescence output of the cells (Figure 5d,e). Together, these findings demonstrated that PIF4 directly binds to and activates the *BEE2* promoter, suggesting that low R:FR ratios, which allow PIF4 protein accumulation in the absence of Pfr, subsequently activate *BEE2* expression by direct promoter binding.

### *BEE2* promotes fiber elongation by upregulating expression of cell‐wall‐loosening genes

**Figure 6:**
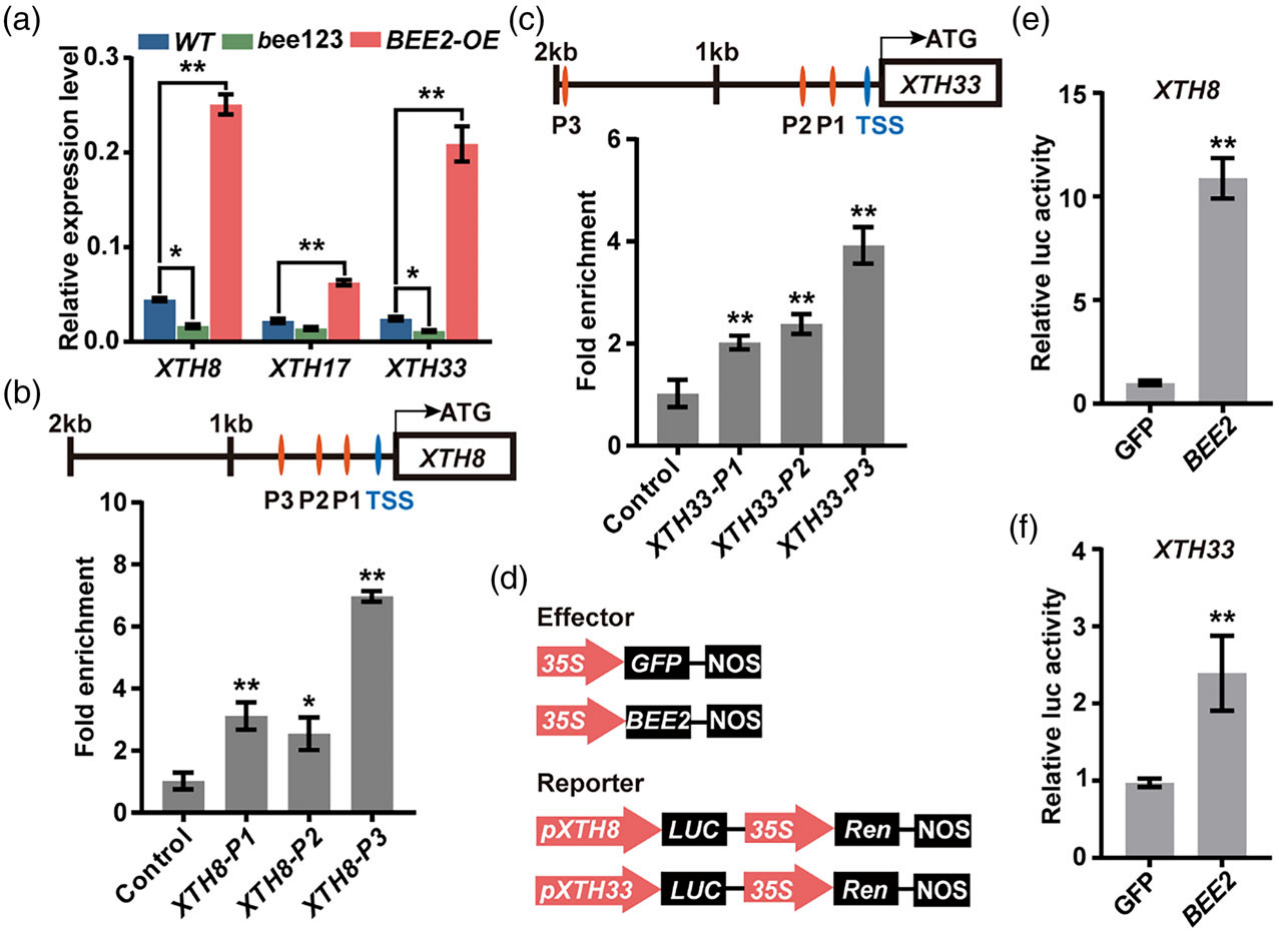
*BEE2* regulates a family of *XTH* genes involved in fiber cell elongation. (a) Relative expression levels of *XTH8*, *XTH17*, and *XTH33* mRNAs in wild‐type (WT), *bee123*, and *BEE2‐OE* plants as determined by quantitative reverse transcriptase‐polymerase chain reaction (qRT‐PCR) using total RNA isolated from 10‐day‐old seedlings grown under white light (WL). Data are mean (±SD) of three biological replicates normalized to that for *ACT2*. (b) ChIP‐qPCR analysis showing BEE2 protein enrichment at the P1, P2, and P3 regions of the *XTH8* promoter. Data represent the mean (±SD) of technical replicates from a representative experiment. Similar results were obtained in three independent experiments. The BEE2 protein was expressed with a 3xHA tag; see Figure S3 for its levels of expression *in planta*. The orange ovals in the gene diagrams in panels (b) and (c) identify the positions of the predicted G‐box‐like elements. The predicted transcription start sites (TSS) and translation start code (ATG) are indicated. (c) ChIP‐qPCR analysis showing enrichment of the BEE2‐HA protein at the P1, P2, and P3 G‐box‐type elements. Data represent the mean (±SD) of technical replicates from a representative experiment. Similar results were obtained in three independent experiments. (d) Schematic representation of the dual‐luciferase reporter constructions (*pXTH8*::LUC/REN and *pXTH33*::LUC/REN) and effector construction (*35S::BEE2*) used for transient expression assays in Arabidopsis protoplasts. (e and f) The BEE2 protein activates the *XTH8* (e) and *XTH33* (f) promoters in Arabidopsis protoplasts. Protoplasts from rosette leaves were co‐transformed with the reporter constructions together with the BEE2 effector construction. After transfection, the protoplasts were kept in the dark for 16 h. Relative luciferase activity (LUC/REN) was normalized to that in protoplasts transformed with the reporter alone and the empty effector (GFP). Data represent mean (±SD) of three biological replicates. * Indicates significant differences at *P* < 0.05, and ** indicates *P* < 0.01 as determined by the Student's *t*‐test for the comparisons shown in panels b, c, e and f.

Key to fully understanding how the PhyB/PIF4 module coupled to *BEEs* expression controls stem elongation would be the identification of the transcriptional targets of BEE2. Obvious possibilities are *the XTH* genes proposed to encode cell‐wall‐loosening factors that could contribute to cell elongation (Eklöf & Brumer, 2010). Arabidopsis encodes 33 *XTH* paralogs, of which *XTH8*, *XTH17*, and *XTH33* were of particular interest because our prior transcriptome analyses and subsequent qRT‐PCR validations revealed that their expression was significantly induced under low R:FR ratios (Figure 2b; Figure S1C). qRT‐PCR analyses conducted here for the *XTH8*, *XTH17*, and *XTH33* mRNAs showed that they are expressed across many tissues, especially in elongating stems, with *XTH8* and *XTH33* showing relatively higher transcript levels (Figure S6A), consistent with their proposed roles in promoting cell expansion (Eklöf & Brumer, 2010). Given the positive role of BEE2 in stem elongation, we tested whether these *XTH* genes are regulated by this transcription factor. qRT‐PCR analyses revealed that *XTH8*, *XTH17*, and *XTH33* expression was significantly upregulated in *BEE2‐OE* plants but downregulated in the *bee123* triple mutant as compared to those in WT (Figure 6a), suggesting that BEE2 promotes stem elongation, at least in part, by transcriptionally activating the expression of these *XTH* genes.

Notably, inspection of the *XTH8* and *XTH33* promoters also identified three possible G‐box‐like *cis‐*elements within 2‐kb and 700‐bp of their transcription start sites, respectively (Figure 6b,c), which could be BEE2‐binding sites based on their known preference for such elements (Friedrichsen et al., 2002). Sequence analysis revealed that the P1–P3 elements in the *XTH8* promoter correspond to CACTTG (P1), CACTTG (P2), and CACGTG (P3), while those in the *XTH33* promoter correspond to CACATG (P1), CACATGCAAATG (P2), and CACGTG (P3) (Figure S6B). Overall, these elements more closely match the canonical G‐box sequence (CACGTG) than those identified in the *BEE* promoters.

ChIP‐qPCR assays using the BEE2‐HA protein as a probe provided support for this binding at both *XTH* promoters with significant enrichment of DNA fragments associated with BEE2 at all sites examined (Figure 6b,c). Similarly, dual‐LUC assays using Arabidopsis protoplasts transfected with the *35S::BEE2‐HA* effector and the *proXTH8::LUC* or *proXTH33::LUC* reporters showed that BEE2 strongly activates the *XTH8* and *XTH33* promoters, with LUC activity increased by approximately 11‐fold and approximately 2.4 fold, respectively, as compared to a GFP control (Figure 6d,f). Together, these findings demonstrate that BEE2 can directly activate *XTH8* and *XTH33* transcription. Given that PIFs also recognize the G‐box motif, which exists in the *XTH8* and *XTH33* promoters. We examined whether PIFs can interact with the *XTH8* and *XTH33* promoters using ChIP assays. It appeared that PIFs showed little interaction with the *XTH8* and *XTH33* promoter fragments in *planta* (Figure S7A,B). The results suggest that PIFs do not directly activate *XTH8* and *XTH33* expression, but instead use the BEE1‐3 intermediates.

**Figure 7:**
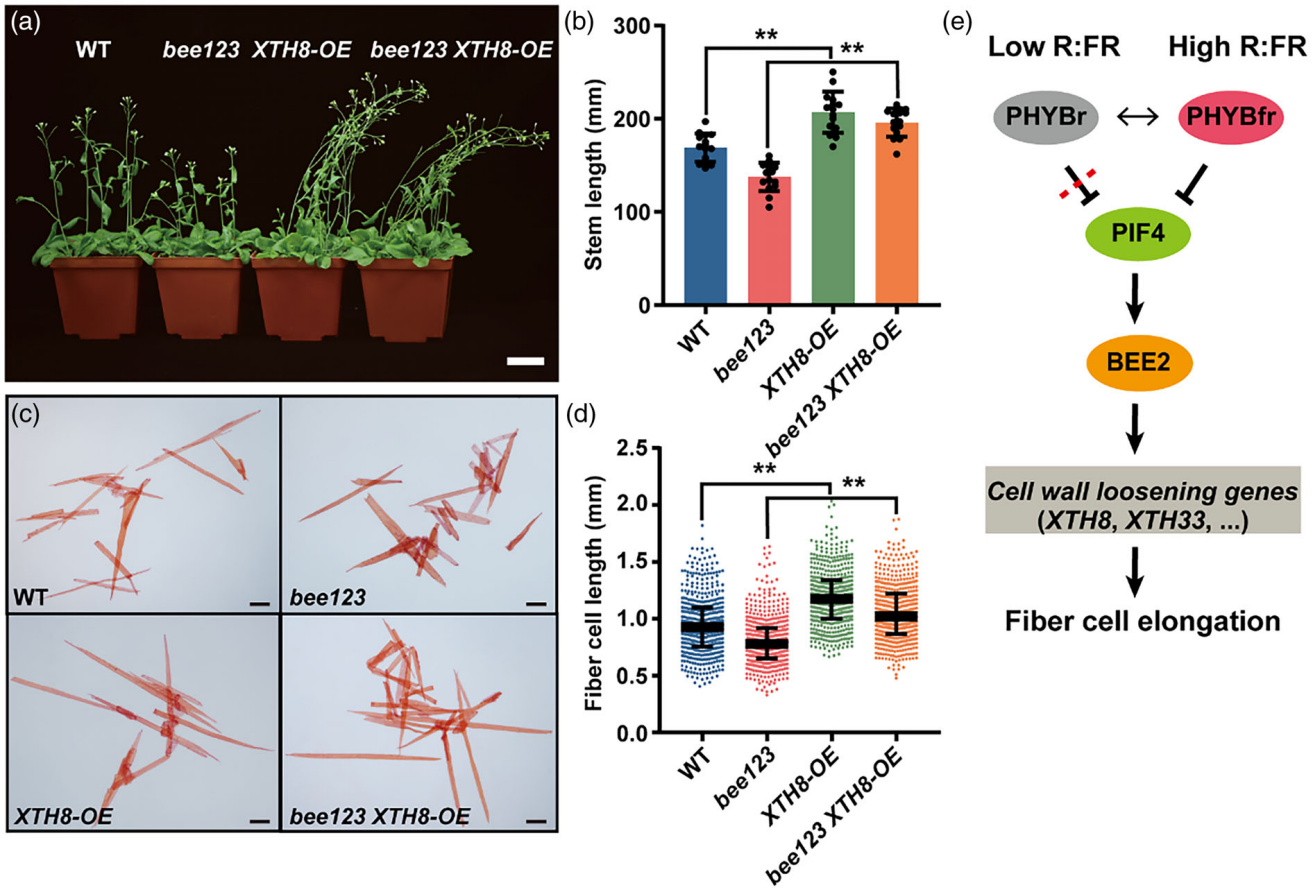
BEEs genetically modulate stem fiber elongation by activating the expression of cell wall‐loosening genes. (a) Photographs of wild‐type (WT), *bee123*, *XTH8‐OE*, and *bee123 XTH8‐OE* Arabidopsis plants grown under white light (WL). Scale bar = 5 cm. See Figure S8 that confirms the expression of the *XTH8* mRNA and protein tagged in‐frame with yellow fluorescence protein (YFP) in the *XTH8‐OE* backgrounds. (b) Bar graphs showing the mean length of WT, *bee123*, *XTH8‐OE*, and *bee123 XTH8‐OE* plants grown as shown in panel (a). Data represent the mean (±SD) of 15 seedlings. ** Indicates significant differences as determined by the Tukey's HSD test (*P* < 0.01) in panels (b and d). (c) Photographs of representative fiber cells extracted from the inflorescence stems of 4‐week‐old WT, *bee123*, *XTH8‐OE*, and *bee123. XTH8‐OE* plants, stained with Safranin. Scale bars = 0.2 mm. (d) Scatter plots showing the distribution of fiber cell lengths in WT, *bee123*, *XTH8‐OE*, and *bee123 XTH8‐OE* inflorescence stems. Data represent mean (±SD) of three biological replicates, each containing >100 cells. (e) A proposed model for the PhyB/PIF‐mediated photoregulatory module controlling fiber cell elongation in Arabidopsis. The R:FR signal is sensed by PhyB, whose Pfr state binds to the PIF transcription factors leading to the mutual degradation of both proteins. In the absence of this proteolysis in the dark or under low R:FR ratio light conditions when Pfr levels are low, PIFs bind to and induces the expression of the family of BEE transcription factors in inflorescence stems. The BEE2 protein, and likely other members of the family, subsequently promotes the expression of genes related to stem growth by direct binding to their G‐box‐type promoter elements, including the *XTH8* and *XTH33* genes encoding xyloglucan endotransglycosylases that help break down cell‐wall interconnections. These XTHs then promote cell‐wall loosening and ultimately fiber cell elongation during accelerated inflorescence stem growth under low R (i.e., low Pfr) conditions.

*XTH8*, which showed a relatively high level of expression in elongating stems (Figure S6A), was selected to directly test for a role for the XTH pair in stem fiber elongation. Here, we generated an *XTH8*‐overexpression line using the full‐length mRNA tagged in‐frame with that for yellow fluorescence protein (YFP) whose expression was driven by the constitutive *35S* promoter in both WT (*XTH8‐OE*) and the *bee123* mutant (*bee123 XTH8‐OE*) backgrounds (Figure S8). Immunoblot analyses with anti‐GFP antibodies subsequently detected the XTH8‐YFP protein in both lines. Transgenic *XTH8‐OE* plants grew significantly longer stems as compared to those of WT, whereas the overexpression of *XTH8* in the *bee123* background effectively rescued the short‐stem phenotype (Figure 7a,b). Consistent with these morphological changes, stem fiber cell length was significantly increased in the *XTH8‐OE* plants (1.3‐fold) and was largely restored back to WT lengths in the *bee123 XTH8‐OE* plants relative to *bee123* stems (Figure 7c,d). XTH8‐YFP overexpression also increased the frequency of inflorescence lodging (Figure 7a), consistent with previous reports linking XTH activity, and possibly those of other xyloglucan endotransglycosylases/hydrolases, to weakened stem strength as stem fibers hyperelongate (Kushwah et al., 2020). Collectively, these results support a model in which BEE2 promotes stem and fiber cell elongation through PhyB/PIF signaling primarily by directly activating expression of downstream genes associated with cell‐wall remodeling, such as *XTH8 and XTH33* when Pfr levels are low.

## DISCUSSION

Stem elongation during plant growth is driven by proliferation of the shoot apical meristem and elongation of the resulting stem cells, which is primarily directed by enzymes that loosen the surrounding primary cell walls, followed by turgor‐driven cell expansion and subsequent rigidification of the secondary cell walls (Hilty et al., 2021; Miao et al., 2024). Here, we showed that the PhyB/PIF4 module plays a key photoregulatory role in stem elongation in part through its impact on this process in fiber cells associated with the vascular tissue. Notably, while most studies of phyB/PIF‐mediated shade avoidance have focused on hypocotyl elongation, ours is one of the first to concentrate on inflorescence stem elongation during the shade avoidance of adult plants. In the canonical light signaling pathway, PhyB as Pfr suppresses elongation under high R:FR conditions by binding to the family of PIF transcriptional activators/repressors which then promotes their mutual degradation. In darkness or under low R:FR light when there is little to no Pfr present, this degradation is disabled, thus allowing PIFs to impact their associated genes. For inflorescence stems and juvenile hypocotyls, this leads to accelerated cell elongation (especially fibers and vascular cells associated with xylem and phloem), which are hallmarks of the shade‐avoidance syndrome (Casal, 2012; Pham et al., 2018).

During our study on this stem elongation process, we found through RNA‐seq analysis that the PhyB/PIF4 module in the absence of Pfr up‐ and down‐regulates a battery of genes associated with cell‐wall biogenesis and loosening, and cell elongation, as well as identified the three‐member BEEs bHLH transcription factor family as being strongly upregulated, thus further connecting light and BR signaling to fiber elongation. These results were similar to earlier RNA microarray studies that also discovered the upregulation of numerous cell‐wall remodeling factors associated with shade avoidance under low Pfr levels (Devlin et al., 2003). Although PIFs are generally regarded as repressors of photomorphogenesis under normal light conditions, they function during shade avoidance, in part, as transcriptional activators that induce *BEE* expression to drive a skotomorphogenesis elongation program under darkness and low R:FR conditions.

Several previous ChIP‐seq and RNA‐seq studies had identified the *BEE2* locus as a potential target of PIF3, PIF4, and PIF5 (Hornitschek et al., 2012; Oh et al., 2012; Zhang, Li, et al., 2013). Here, we confirmed that transcript levels of *BEE1*, *BEE2*, and *BEE3* are significantly increased in inflorescence stems in response to low R:FR light. This activated expression was directly caused by binding of PIF4 to G‐box‐like elements within the *BEE* promoters, which we presume accelerates their expression. Given the proposed roles of XTHs in cell‐wall loosening through their xyloglucan endotransglycosylases/hydrolase activities and prior studies connecting them to cell‐wall loosening in trees (Cosgrove, 2024; Eklöf & Brumer, 2010), we then assayed for their regulation by BEEs and found that their expression is indeed controlled by both the PhyB/PIF4 module and the BEEs regulators. In this way, we have identified a regulatory circuit starting from light and ending at loosened cell walls associated with shade avoidance.

Based on this regulatory framework, we propose a molecular model for light‐regulated stem fiber cell elongation (Figure 7e). Under high R:FR conditions, PhyB in its Pfr form promotes PIF degradation, thereby repressing elongation‐related gene expression. In contrast, under darkness or low R:FR light, reduced Pfr levels (Casal, 2012) allow PIFs to accumulate and induce expression of the *BEE* transcription factors in inflorescence stems. BEE2, together with other BEE family members, then activates downstream genes, such as *XTH8* and *XTH33*, potentially promoting cell‐wall loosening and ultimately fiber cell elongation during shade avoidance. While it is likely that all these factors interact with each other directly and function specifically in fiber cells, we acknowledge that other stem cell types could also be involved. Intercellular communication plays a role in coordinating stem elongation. Previous studies have shown that PHYB in epidermal cells can influence the growth of inner tissues, including the endodermis and hypocotyl, through an as‐yet unidentified mobile signal (Kim et al., 2016). In addition, our GUS analyses indicate that *BEE2* is expressed in both cortical and fiber cells in elongating stem. Taken together, these observations suggest that the light‐regulated circuit identified here also likely operates in multiple stem tissues and cell types to enable a coordinated growth response.

As such this R → PhyB→PIF4 → BEEs→XTHs→cell wall‐loosening circuit now represents one of the better understood cascades within the Phy signaling system that controls a myriad of light‐regulated process, such as seed germination, tropisms, chloroplast development, flowering time, and senescence (Franklin & Quail, 2010). Other well‐established effector cascades associated with shade avoidance include the PIF‐dependent activation of the auxin biosynthetic gene *YUCCA8* (Sun et al., 2012), the PIF‐mediated cooperative regulation of growth and stress responses through interactions with the BRASSINAZOLE‐RESISTANT 1 (BZR1) and MYC DOMAIN PROTEIN 2 (MYC2) transcription factors (Oh et al., 2012; Zhao et al., 2021), and the PIF‐driven modulation of the B‐BOX DOMAIN PROTEIN (BBX) transcription factor family (Zhang et al., 2017). Despite this complexity, most other PhyB/PIF‐regulated circuits involve extensive hormonal amplification and transcriptional networks, and only rarely has a continuous regulatory cascade been defined that directly links light perception to downstream effector enzymes. By contrast, the PhyB/PIF–BEE2–XTH circuit described here represents a relatively simple and mechanistically well‐resolved regulatory cascade that directly couples light signaling to cell‐wall remodeling, as well as provides a clear example with an applied relevance for understanding how the PhyB/PIF module regulates cell‐type‐specific development under shade.

It has not escaped our attention that the *BEE1‐3* and *XTH8*, *17*, and *33* promoters studied here all harbor similar, but not identical, strings of G‐Box‐type motifs (mainly C‐A‐C/T‐G/A‐T‐G) that appear to bind the PIF and BEEs transcription factors, respectively (Figures S4A and S5B). Consistent with this observation, we examined the relationship between PIFs and *XTH* promoters and found that PIFs do not directly bind to the *XTH* promoters in *planta* (Figure S7). These results suggest that PIFs activate *XTH* expression indirectly through BEE‐mediated hierarchical regulation. These findings raise the possibility that subtle, as‐yet‐unappreciated differences in these G‐box‐like sequences may provide sufficient specificity for selective PIF and BEE binding, thereby supporting their distinct target specificities. Clearly, additional studies are needed to determine how the PIF → BEE cascade identified here is integrated with responses in other cell types (e.g., epidermis) in stems beyond its role in regulating fiber cell responses.

A wealth of genetic and transcriptomics have implicated a myriad of transcriptional regulators, effector proteins, and enzymes that contribute to the light responses triggered by photoactivated Phys, including both positive and negative regulators (Huq et al., 2024). For the switch from skotomorphogenesis to photomorphogenesis and the deactivation of the associated shade‐avoidance response, it is notable here that constitutive over‐ or under‐expression of a single BEE transcription factor (BEE2) and overexpression of a single xyloglucan endotransglycosylase/hydrolase‐XTH8 (of 33 total possible paralogs), which is controlled by BEE2 through PhyB/PIF4, were able to replicate, for the most part, the effects of PhyB on stem elongation and associated fiber cell length (see Figure 7). Consequently, it argues that this PhyB‐regulated, shade‐avoidance process involves a subset of key regulators and associated enzymes. Therefore, it is conceivable that the modification through light signaling of fiber properties for agricultural benefit might be more simple than previously realized, involving a limited number of core components working in short cascades. The R → PhyB/PIF4 → BEEs→XTHs→loosened cell walls module might be just one of several examples of this simplicity. Other recent examples include the transgenic overexpression of the expansin *EXPA1* to increase tree height and fiber cell diameter (Gray‐Mitsumune et al., 2004, 2008), and the fiber‐specific upregulation of the aspen cellulase Cel9A6 to increase fiber cell length (Li et al., 2025; Yu et al., 2013).

In angiosperms, fiber cells, which provide mechanical support for stem growth, offer a renewable supply for human‐required fiber materials. Wood fiber in particular is widely used in the pulp and papermaking industries where fiber cell characteristics, including length and cell‐wall thickness, and chemical composition dictate their utility (Madsen & Gamstedt, 2013; Rana et al., 2014). Multiple studies have shown that the genetic modification of fiber cells in plant stems could have economic benefit. Examples include silencing GA 2‐oxidase to enhance fiber production (Dayan et al., 2010), the suppression of *4CL* gene expression in stem fibers to reduce lignin levels (Cao et al., 2020), the precise modulation of the LTF1 transcription factor in stem fiber cells to diminish lignin content without impacting field growth (Gui et al., 2020), and methods to increase the length of stem fiber to improve pulping efficiency and paper performance (Li et al., 2025). This study reveals that modification of light signaling through the PhyB/PIF4 module in conjunction with BEE2 and XTHs also offers new strategies to modify fiber cell properties through genetic modification of photoregulatory modules and downstream effectors, with the *proviso* that stem strength can be sufficiently maintained to prevent lodging. These insights open opportunities to develop plants for enhance fiber production and utilization.

## MATERIALS AND METHODS

### Plant materials and growth conditions

WT plants and related mutants and transgenic materials used in this study were derived from the *A. thaliana* Columbia‐0 (Col‐0) ecotype. The *phyB*, *pifq*, *PIF4‐OE*, and *bee123 triple mutant*s were described previously (Luo et al., 2022; Wang et al., 2019). Other overexpression lines were generated by transforming WT or mutant seedlings with engineered transgenes using the floral dip method (Clough & Bent, 1998), which involves briefly immersing the inflorescences of bolting plants in an *Agrobacterium*‐containing infiltration solution, followed by a 24‐h dark treatment before returning the plants to normal growth conditions. After germination of the resulting seeds, the F1 progenies were screened by genomic PCR to identify successful transformants, and qRT‐PCR and/or immunoblot assays were subsequently performed to identify lines with sufficient expression levels. Homozygous plants bearing the mutations and/or transgenes were identified in the F2 progeny by genomic PCR. Plants were grown in controlled environment chambers at 22°C under a 16‐h light/8‐h dark photoperiod provided by 80 μmol m^−2^ sec^−1^ WL. At emergence of the inflorescence stem, the plants were introduced to WL + FR light conditions to alter stem growth. The intensity of the WL was 67 μmol m^−2^ sec^−1^, whereas the amended FR at 720–750 nm was emitted by a FR LED (Qiding Photoelectric Company Limited, Shanghai) with an intensity of 47 μmol m^−2^ sec^−1^. All light parameters were measured with an ILT1400 Radiometer Photometer and an Ocean Optics HR2000 + CG spectrophotometer.

### Construction of vectors and generation of transgenic plants

All primers used to assemble the various DNA constructions are listed in Table S2. A 912‐bp coding sequence of *BEE2* was introduced into the *pCAMBIA1300‐35Spro‐BEE2‐3HA* vector for the CaMV *35S*‐driven overexpression of the BEE2 protein in WT Arabidopsis. The full‐length *BEE2* coding sequence was first PCR‐amplified with primers carrying *Kpn*I and *Bam*HI restriction sites, digested with these enzymes (Thermo Fisher, Waltham, MA, USA), and then ligated into the *Kpn*I/*Bam*HI‐linearized pCAMBIA1300‐35Spro‐3HA backbone to introduce an in‐frame C‐terminal 3xHA tag. *pifq BEE2‐OE* plants were generated by transforming the *pCAMBIA1300‐BEE2‐3HA* construction into the *pifq* mutant. To constitutively increase *XTH8* expression in Arabidopsis, a 915‐bp fragment of the full‐length *XTH8* coding sequence was cloned into the *pHB‐X‐YFP* vector in‐frame with the YFP coding sequence using Gibson assembly (Gibson et al., 2009), thereby generating the *pHB‐35Spro‐XTH8‐YFP* construction, which was then transformed into WT plants. The *bee123 XTH8‐OE* line was generated by transforming the *bee123* mutant with the *pHB‐35Spro‐XTH8‐YFP* vector.

### Stem fiber analysis

Fiber cell extraction and staining were performed as previously described (Zhang et al., 2018). When Arabidopsis inflorescence stems reached their full height, basal 2‐cm segments were dissected, cut into small pieces, and dissolved in a 1:1 (v/v) glacial acetic acid/30% hydrogen peroxide mixture. The reactions were then incubated at 80 °C for 12–16 h with intermittent shaking to release fiber cells. Fibers were stained with 1% Safranin (Solarbio) for 10 min, a basic dye that preferentially stains red lignified secondary cell walls, washed twice with H_2_O, and observed under an optical microscope (Olympus BX53). Lengths of the fiber cells, identified based on their characteristic morphology as long, slender cells with tapered ends, were quantified by ImageJ (https://imagej.nih.gov/ij/download.html).

### RNA isolation and transcript analysis

Total RNA was extracted from various tissues of *A. thaliana* Col‐0 utilizing the E.Z.N.A. Plant RNA Kit (R6827‐02; OMEGA, Norcross, GA, USA) following the manufacturer's directions. RNA quality and concentration were assessed by a NanoDrop spectrophotometer (NanoDrop Technologies, Wilmington, DE, USA). First‐strand cDNA was synthesized from 1 μg of total RNA utilizing the TransScript One‐Step gDNA Removal and cDNA Synthesis SuperMix Kit (AT311‐03; TransGen Biotech, Beijing, China), and the resulting cDNA was diluted 25‐fold prior to qPCR. qRT‐PCR was performed with a QuantStudio 3 real‐time PCR system (Applied Biosystems, Foster City, CA, USA) in 20‐μL reactions containing 10 μL of SYBR Green Master Mix (AQ132‐21; TransGen Biotech), 0.5 μL of each primer (see Table S2), 1 μL of nuclease‐free water, and 8 μL of diluted cDNA. Each condition/genotype was tested by three biological replicates each assayed by three technical replicates. Relative expression levels were calculated from the average values using the 2^−ΔΔCt^ method, with results from the *ACT2* (At3g18780) mRNA serving as the internal reference.

### RNA‐seq analysis

For RNA‐seq, WT Col‐0 plants were grown in WL in the greenhouse until emergence of the inflorescence stem, after which they were subjected to low R:FR ratio light for 10 d. Total stem RNA was isolated from three biological replicates of WT and assessed for RNA integrity by an Agilent 2100 Bioanalyzer, with the RNA concentrations then measured using a NanoDrop ND‐2000 spectrophotometer (NanoDrop Technologies). Two micrograms of total RNA per sample were employed to assemble strand‐specific cDNA libraries using the TruSeq Stranded mRNA LT Sample Prep Kit (Illumina). The libraries were size‐selected for fragments of approximately 300‐bp in length by electrophoresis in 2% low‐range ultra‐agarose and then PCR‐amplified with the Phusion DNA polymerase (Thermo Fisher) for 15 cycles.

After quantification, the paired‐end libraries were sequenced in an Illumina HiSeq X10 platform at Oebiotech Corporation (Shanghai, China). Raw paired‐end reads were trimmed and quality controlled using SeqPrep (https://github.com/jstjohn/SeqPrep) and Sickle (https://github.com/najoshi/sickle) with default parameters used to remove adapter contamination and low‐quality bases. Clean reads were then aligned to the *A. thaliana* TAIR10 reference genome using the vHISAT2 software (https://daehwankimlab.github.io/hisat2/) in the appropriate orientation mode. Mapped reads were assembled in a reference‐guided manner by StringTie (https://ccb.jhu.edu/software/stringtie/). Gene expression levels were quantified by RSEM (http://deweylab.biostat.wisc.edu/rsem/) to obtain both raw read counts for differential expression analysis, and transcripts per million (TPM) values normalized for gene length and sequencing depth for expression visualization.

DEGs between WL and low R:FR ratio samples were identified using DESeq2 (Love et al., 2014), with significantly regulated genes defined as those with |log_2_FoldChange| ≥1 (corresponding to a fold change ≥2) and an adjusted *P*‐value (q‐value) <0.05. Volcano plot, Heatmap, and KEGG pathway enrichment analyses of the DEGs was performed using the online bioinformatics platform available at https://www.bioinformatics.com.cn/.

### Immunoblot analysis

Total soluble protein was extracted from the aerial parts of 10‐day‐old Arabidopsis grown under WL on Murashige and Skoog (MS) medium plus 2% (w/v) sucrose. The shoots were frozen in liquid nitrogen, pulverized using a bead mill combined with stainless‐steel beads, and homogenized in extraction buffer (20 mM Tris–HCl (pH 7.5), 150 mM NaCl, 5 mM MgCl_2_, 0.5% (v/v) NP‐40, and 1× protease inhibitor cocktail). After clarification at 12 000 g for 10 min at 4°C, the supernatant was mixed with 5 × SDS loading buffer and heated to 100°C for 10 min, clarified by centrifugation, and used directly for SDS–PAGE.

Proteins (20–40 μg) were electrophoresed in 4–20% acrylamide SDS–PAGE gels and electrophoretically transferred onto methanol‐activated PVDF membranes (Bio‐Rad). Membranes were blocked with 5% (w/v) skimmed milk (S39343‐250G; ABCONE, Shanghai, China) in Tris‐buffered saline containing Triton X‐100 (TBST; 20 mM Tris–HCl (pH 7.5), 150 mM NaCl, and 0.1% (v/v) Triton X‐100) and incubated overnight with anti‐HA/GFP (1:2000; Abmart) or anti‐Actin (1:2000 dilution; Abmart, Shanghai, China) antibodies diluted in TBST. After washing, the membranes were incubated with a horseradish peroxidase (HRP)‐conjugated goat‐anti‐mouse secondary antibody (1:5000 dilution; Thermo Fisher) for 1 h and then rewashed for an additional 1 h in TBST. Immunoblot signals were detected using the Tanon™ High‐sig Enhanced Chemluminescence (ECL) Western Blotting Substrate (180‐501; Tanon, Shanghai, China) and photographed with the Tanon Imaging System (5200CE; Tano).

### Yeast one‐hybrid assay

Y1H analyses were performed as previously described (He et al., 2025). Briefly, approximately 575‐bp genomic fragment of the *BEE2* promoter ending at the translation start site and containing the P2 *cis*‐element was subcloned into the pAbAi vector (Takara) through *Hind*III and *Xho*I restriction digestion and ligation, whereas the full‐length *PIF4* coding sequence was cloned into the pGADT7 vector (Takara) via Gibson assembly. The *pAbAi::BEE2* construction was introduced into the *Saccharomyces cerevisiae* strain Y187 cells to generate the bait using the manufacturer's instructions for the Matchmaker Gold Yeast One‐Hybrid System (Takara). Their self‐activation activity was tested on SD‐Ura medium containing 200, 400, or 800 ng/mL of AbA. For interaction assays, the *pAbAi::BEE2* and *pGADT7::PIF4* plasmids were co‐transformed into Y187 cells using the standard lithium acetate (LiAc)/polyethylene glycol (PEG) yeast transformation protocol, with co‐transformation of *pAbAi::BEE2* with the empty *pGADT7* vector serving as the negative control. Y187 bait cells were grown in Yeast Extract‐Peptone‐Dextrose‐Adenine (YPDA) medium to mid‐log phase, converted to competent cells with Tris‐EDTA buffer (TE)/LiAc (10 mM Tris–HCl (pH 7.5), 1 mM Na_2_EDTA, and 100 mM lithium acetate), and mixed with plasmid DNA and denatured carrier DNA, followed by incubation with LiAc/PEG solution (40% (w/v) PEG 4000, 100 mM lithium acetate, 10 mM Tris–HCl (pH 7.5), and 1 mM Na_2_EDTA). After heat shock at 42°C, the cells were recovered in 0.9% (w/v) NaCl and plated onto SD/−Leu medium to select for AD‐plasmid transformants. Single positive colonies were then replica‐plated onto SD/−Leu plates supplemented with 800 ng/mL AbA and incubated at 30°C for 3–5 days to assess *BEE2* promoter–dependent reporter activation.

### Dual‐luciferase reporter assay

For the dual‐LUC assays, the full coding sequences from Arabidopsis *PIF4* and *BEE2* were introduced into the pA7 vector using the *Kpn*I and *Spe*I restriction sites to generate the effector constructions *pA7‐35S::PIF4* and *pA7‐35S::BEE2*. The *BEE2*, *XTH8*, and *XTH33* promoters were inserted upstream of the coding region for the LUC reporter in the pGreenII 0800‐LUC vector by Gibson assembly, with the REN coding sequence serving as an internal control. Arabidopsis protoplast isolation and PEG‐mediated transformation were performed as previously described (Luo et al., 2022). LUC and REN activities were quantified using the Dual‐Luciferase Reporter Assay Kit (E1910; Promega) in combination with a GloMax 20/20 Luminometer (Promega, Madison, WI, USA).

### Electrophoretic mobility shift assays

Recombinant PIF4 was purified and subjected to EMSA as previously described (Luo et al., 2022; Wang et al., 2019). The PIF4 coding sequence was inserted downstream of the *E. coli* cspA cold‐shock promoter in the pCold‐TF vector (Takara) using the *Nde*I and *Xba*I restriction sites, ensuring that the N terminus of PIF4 remained in‐frame with the N‐terminal Trigger Factor (TF) and 6xHis fusion sequences (HHHHHH). After transforming the construction into *BL21* (DE3) cells, protein expression was induced overnight at 16°C with 0.5 mM isopropyl β‐D‐1‐thiogalactopyranoside (IPTG). The PIF4–6xHis protein was purified using Ni‐NTA agarose resin with 250 mM imidazole used for elution (30 210; Qiagen, Shanghai, China). DNA probes corresponding to the designed *BEE2* promoter fragments were PCR‐amplified from genomic DNA, and mutated probes were generated by PCR using mutation‐specific primers. All amplified fragments were cloned into the pEASY‐Blunt vector (TransGen) and used as templates for probe preparation. Cy5‐labeled probes were generated by PCR using M13F‐Cy5 and M13R primers, whereas unlabeled competitor probes were amplified using unlabeled M13F and M13R primers (Table S2).

EMSA reactions (20 μL) contained 20 mM Tris–HCl (pH 7.9), 5% (v/v) glycerol, 0.04 mg/mL BSA, 0.5 mM MgCl_2_, 0.1 mM DTT, 2 μg salmon sperm DNA, 10 ng Cy5‐labeled DNA probe, and purified 6xHis‐PIF4 protein, with water added to a final volume of 20 μL. Reaction mixtures were incubated at room temperature for 20 min to allow protein‐DNA complex formation. The complexes were then resolved on 4.5% (w/v) native polyacrylamide gels in 0.5xTBE buffer (45 mM Tris‐borate (pH 8.3) and 1 mM Na_2_EDTA), which had been exposed for 1 h at 100 V on ice. Samples were electrophoresed at 100 V for 1–2 h at low temperature in the dark, and Cy5 fluorescence was detected using an FLA 9000 imaging system (Fujifilm, Tokyo, Japan).

### Chromatin immunoprecipitation PCR assay

For immunoblot analysis, the PIF4‐OE, BEE2‐OE, and XTH‐YFP proteins were expressed in Arabidopsis plants and detected by immunoblotting of total seedling extracts with anti‐MYC (Abmart, for PIF4), anti‐HA (Abmart, for BEE2), and anti‐GFP antibodies (Abmart, for GFP), respectively, coupled with HRP‐conjugated anti‐mouse IgGs as the secondary antibody. Actin served as the loading control and was detected with anti‐Actin antibodies (Abmart).

For ChIP analysis, T2 seedlings expressing PIF4‐MYC or BEE2‐HA were used. Chromatin immunoprecipitation was performed essentially as described (Bowler et al., 2004) using a Magna ChIP A/G Kit (Millipore). Briefly, 10‐day‐old seedlings grown on MS medium supplemented with 2% (w/v) sucrose were cross‐linked under a vacuum in 1% (v/v) formaldehyde and quenched with 0.125 M glycine. Chromatin from isolated nuclei was fragmented using a Covaris E220 AFA sonicator (Covaris Inc.) to yield DNA fragments of approximately 200–500 bp in length. Chromatin complexes were captured by immunoprecipitation with protein A/G magnetic beads coated with anti‐MYC or anti‐HA antibodies (Sigma). After extensive washing, cross‐links were reversed, and the released DNA was purified according to the manufacturer's instructions (Millipore). Enriched DNA fragments were quantified by qPCR using the SYBR Green Master Mix (AQ132‐21; TransGen Biotech), with the *ACT2* gene serving as the internal reference. Fold enrichment was calculated relative to input DNA.

### Promoter‐GUS histochemical staining

The *BEE2* promoter (−1 to −2906 bp from ATG start codon) was cloned from *A. thaliana* Col‐0 DNA using Gibson assembly into the pORE‐R2 vector upstream of the full GUS coding region (Luo et al., 2022), which was then transformed into WT Arabidopsis by the floral dip method (Clough & Bent, 1998). Transgenic *BEE2pro‐GUS* plants were selected on MS medium containing 50 μg/mL hygromycin and then genotyped as correct by PCR using GUS‐specific primers. Positive T2 transgenic plants were used for analysis of GUS activity. Stem segments from the middle region of elongating stems of 5‐week‐old plants were hand‐sectioned using a razor blade. The samples were pretreated with acetone for 10 min at 4°C, washed three times with 100 mM sodium phosphate (pH 7.0), and incubated in GUS staining solution (100 mM sodium phosphate (pH 7.0), 10 mM Na_2_EDTA, 2 mM 5‐bromo‐4‐chloro‐3‐indolyl‐β‐D‐glucuronic acid, 5 mM K₄Fe(CN)₆, 5 mM K₃Fe(CN)₆, and 0.2% Triton X‐100) at 37°C for to 3 h. Stained samples were observed under an optical microscope (Olympus BX53).

## GENE ACCESSION NUMBERS

Sequence data from this article can be found at https://www.arabidopsis.org with the following accession numbers: *PHYB* (At2g18790), *PIF1* (At2g20180), *PIF3* (At1g09530), *PIF4* (At2g43010), *PIF5* (At3g59060), *BEE1* (At1g18400), BEE2 (At4g36540), BEE3 (At1g73830), *XTH8* (At1g11545), XTH17 (At1g65310), XTH33 (At1g10550), and *ACT2* (At3g18780).

## AUTHOR CONTRIBUTIONS

FL and LL designed the research. HX, FL, DZ, and XG performed the experiments. HX, FL, and XG analyzed results. HX, FL, RV, and LL discussed the data and wrote the paper. All authors read and approved the article.

## CONFLICT OF INTEREST

The authors declare no competing interests.

## Supporting information


**Figure S1.** Low R:FR condition induces the expression of genes for cell elongation.
**Figure S2.** GUS staining of the *BEE2* promoter in elongating Arabidopsis stems.
**Figure S3.** Overexpression of *BEE2* in WT Arabidopsis.
**Figure S4.** Overexpression of *BEE2* in the *pifq* mutant.
**Figure S5.** PIF4 directly binds to the *BEE2* promoter.
**Figure S6.** Expression of *XTHs* in various tissues of Arabidopsis.
**Figure S7.** Analysis of the PIF4 binding to the *XTH8* and *XTH33* promoters in *planta*.
**Figure S8.** Overexpression of *XTH8* in the *bee123* triple mutant.

**Table S1.** Differentially expressed genes in the transcriptomes of wild‐type Arabidopsis under WL or WL + FR condition.
**Table S2.** List of primers used in this study.

## Data Availability

The data that support the findings of this study are openly available in the National Genomics Data Center at https://ngdc.cncb.ac.cn/gsa, reference number CRA038337.
