# Supplementary material for: The PhyB‐PIF4 module directly photoregulates BR‐ENHANCED EXPRESSION genes to enhance stem fiber cell elongation in Arabidopsis
Source: Plant J. 2026 Sep 14;127(5):e71110. doi: 10.1111/tpj.71110 (PMC13575309; doi:10.1111/tpj.71110)
Supplement: Supplementary file 1 [file TPJ-127-0-s002.docx]

**Supplementary Figures**

**Supplementary Figure S1. Low R:FR condition induces the expression of genes for cell elongation.**

**(A)** Cartoon of the inflorescence stems grown under WL and low R:FR (WL+FR) light conditions used for transcriptome analysis. Total RNA isolated from 4-week-old WT stems was analyzed by RNA-seq using three biological replicates per condition. Comparisons revealed 2,541 differentially expressed genes (DEGs) based on |log₂FC| > 1 and Q-value < 0.05.

**(B)** Volcano plot showing differentially expressed genes (DEGs) detected between WL and WL+FR conditions. Each dot represents an individual gene. A total of 987 genes were significantly upregulated (orange) and 1,464 were significantly downregulated (blue), while gray dots indicate genes without significant changes in abundance.

**(C)** Relative expression levels of the *XTH8*, *XTH17*, and *XTH33* transcripts in inflorescence stems of plants under grown as in panel (A) as determined by qRT-PCR. Bars represent mean (± SD) of three biological replicates normalized to that for *ACT2*. * Indicates significant differences at *p* < 0.05, and ** indicates *p* < 0.01 as determined by Student’s t-test.

**Supplementary Figure S2. GUS staining of the *BEE2* promoter in elongating *Arabidopsis* stems.**

GUS staining of the stem cross-section in the elongating stem from 4-week-old *BEE2pro:GUS* plants. Fi, interfascicular fiber cell; Epi, epidermis; Co, cortex; Ph, phloem; Ca, cambium; Xy, xylem; Ve, vessel element. Scale bar = 100 μm.

**Supplementary Figure S3. Overexpression of *BEE2* in WT *Arabidopsis*.**

**(A)** Relative expression levels of *BEE2* transcripts in WT and *BEE2*-*OE* plants as determined by qRT-PCR using total RNA isolated from 10-day-old seedlings grown under WL. The *BEE2-OE* plants constitutively expressed the *BEE2* mRNA in-frame with that encoding a C-terminal HA tag. Bars represent mean (± SD) of three biological replicates normalized to that for *ACT2*. ** Indicates significant differences as determined by the Student’s *t*-test (*p* < 0.01).

**(B)** Expressed BEE2-HA protein in *BEE2-OE* plants. Total soluble protein extracts were prepared from 10-day-old seedlings and subjected to immunoblot analysis with anti-HA antibodies, using anti-Actin antibodies to confirm near equal loading of the samples. The experiments were repeated twice with similar results.

**Supplementary Figure S4. Overexpression of *BEE2* in the *pifq* mutant.**

**(A)** Relative expression levels of *BEE2* transcripts in WT, *pifq*, *BEE2-OE*, and *pifq BEE2-OE* plants as determined by qRT-PCR using total RNA isolated from 10-day-old seedlings grown under WL. The *BEE2-OE* plants constitutively expressed the *BEE2* mRNA in-frame with that encoding a C-terminal HA tag. Bars represent mean (± SD) of three biological replicates normalized to that for *ACT2*. ** Indicates significant differences as determined by the Student’s *t*-test (*p* < 0.01).

**(B)** Expressed BEE2-HA protein in *BEE2-OE* and *pifq BEE2-OE* plants. Total soluble protein extracts were prepared from 10-day-old seedlings and subjected to immunoblot analysis with anti-HA antibodies, using anti-Actin antibodies to confirm near equal loading of the samples. The experiments were repeated twice with similar results.

**Supplementary Figure S5. PIF4 directly binds to the *BEE2* promoter.**

**(A)** G-box element sequences designated P1, P2, and P3 were predicted within the *Arabidopsis BEE2* promoter.

**(B)** *In vitro* binding of PIF4 to the *BEE2* promoter fragments (P1, P2, and P3) via EMSA. Recombinant PIF4 tagged with a 6xHis-tag was incubated with 10 ng of ~250-bp oligonucleotide probes from the *BEE2* promoter tagged with Cy5 and subjected to gel electrophoresis. Presence of the probes was detected by fluorescence at 670 nm. Locations of the free probe and the PIF4: probe complexes are indicated. EV, empty vector.

**Supplementary Figure S6. Expression of *XTHs* in various tissues of *Arabidopsis*.**

**(A)** Relative expression levels of *XTH8*, *XTH17*, and *XTH33* transcripts in various tissues of 4-week-old *Arabidopsis* plants as determined by qRT-PCR. Bars represent mean (± SD) of three biological replicates normalized to that for *ACT2*.

**(B)** G-box element sequences detected in the *XTH8* and *XTH33* promoters.

**Supplementary Figure S7. Analysis of the PIF4 binding to the *XTH8* and *XTH33* promoters in *planta***

(A) Schematic diagram of the *XTH8* promoter showing the P1, P2, and P3 fragments containing G box-like sequences (orange ovals) that were used for ChIP-qPCR analysis. The promoter fragments showed little interaction with PIF4 in *planta*. The predicted transcription start sites (TSS) and translation start code (ATG) are indicated.

(B) Schematic diagram of the *XTH33* promoter showing the P1, P2, and P3 fragments containing G box-like sequences (orange ovals) that were used for ChIP-qPCR analysis. The promoter fragments showed little interaction with PIF4 in *planta*. The predicted transcription start sites (TSS) and translation start code (ATG) are indicated.

**Supplementary Figure S8. Overexpression of *XTH8* in the *bee123* triple mutant.**

**(A)** Relative expression levels of *XTH8* transcripts in WT, *bee123*, *XTH8-OE*, and *bee123 XTH8-OE* plants as determined by qRT-PCR using total RNA isolated from 10-day-old seedlings grown under WL. The *XTH8-OE* plants constitutively expressed the *XTH8* mRNA in-frame with that encoding a C-terminal YFP tag. Bars represent mean (± SD) of three biological replicates normalized to that for *ACT2*. ** Indicates significant differences as determined by the Student’s *t*-test (*p* < 0.01).

**(B)** Expressed XTH8-YFP protein in *XTH8-OE* and *bee123 XTH8-OE* plants. Total soluble protein extracts were prepared from 10-day-old seedlings and subjected to immunoblot analysis with anti-GFP antibodies, using anti-Actin antibodies to confirm near equal loading of the samples. The experiments were repeated twice with similar results.
